# Phosphatase, pseudo-phosphatase, or both? Understanding PRL oncogenicity

**DOI:** 10.1038/s41416-020-01194-9

**Published:** 2020-12-03

**Authors:** Kalle Gehring, Hiroaki Miki

**Affiliations:** 1 Department of Biochemistry and Centre de Recherche en Biologie Structurale, McGill University, Montreal, QC Canada; 2 Department of Cellular Regulation, Research Institute for Microbial Diseases, Osaka University, Suita, Osaka Japan

**Keywords:** Hydrolases, Metastasis, Enzyme mechanisms

## Abstract

Phosphatases of regenerating liver (PRL1–3) are among the most oncogenic protein phosphatases but their mechanism of action is poorly understood. Multiple substrates have been proposed as well as a non-catalytic function regulating magnesium transport. Our recent identification of a catalytically inactive PRL mutant that retains oncogenicity in a mouse model promises to resolve the question of whether PRLs act as phosphatases or pseudo-phosphatases in different cancer models.

## Main

Phosphatase of regenerating liver (PRL) phosphatases (protein tyrosine phosphatases (PTP4A1–3)) were first identified in the regenerating liver and subsequently found to be upregulated in cancer.^1,2^ The majority of studies have identified an involvement of the Akt–phosphatase and tensin homologue (PTEN) pathway, but no consensus has emerged for their substrates or downstream effectors. In 2014, an alternative function of PRLs was proposed as pseudo-phosphatases that bind and regulate the function of an enigmatic family of membrane proteins, CBS domain metal transport mediator (CNNM) proteins.^3,4^ Two groups independently showed that PRL control of intracellular magnesium levels was important for cancer progression and metastasis.

PRLs are members of the large superfamily of the cysteine-based phosphatases that include PTP1b (PTPN1) and PTEN. The catalytic cycle of the phosphatases is characterised by a two-step mechanism that involves formation of a phosphocysteine intermediate that is hydrolysed to regenerate the active enzyme. Uniquely to PRLs, the phosphocysteine intermediate is stable with a half-life of over an hour.^5,6^ PRLs exhibit burst kinetics in which a single round of catalysis is followed by slower steady-state activity. Like many other phosphatases, the PRL catalytic cysteine is also prone to oxidation and can form a disulphide to reversibly inactivate the enzyme.^7^

Crystal structures of PRLs in complex with a cytosolic domain of CNNM proteins show that the PRL catalytic site binds a CNNM aspartic acid residue that acts as a substrate mimic.^5,8,9^ The PRL catalytic cysteine plays a key role in the interaction. Mutations to either alanine or serine block both catalytic phosphatase activity and CNNM binding. The absence of mutants that could distinguish between the two activities meant that it was impossible to unambiguously attribute the effects of PRL expression to either phosphatase or pseudo-phosphatase activity.

**Fig. 1:**
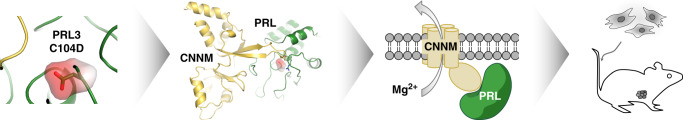
PRL3 acts as a pseudo-phosphatase. Left to right: substitution of the catalytic cysteine 104 by aspartic acid preserves the negative charge required for CNNM binding but abolishes PRL3 catalytic activity. Both wild-type and mutant PRLs can bind CNNM proteins to inhibit magnesium efflux. Analysis of the C104D mutant in the B16 melanoma mouse model shows that CNNM binding is necessary and sufficient for PRL3-driven tumour metastasis.^10^

We recently showed that cysteine substitution by the negatively charged aspartic acid retains CNNM binding.^10^ This initially surprising result reflects the fact that the cysteine residue in PRLs is present as a negatively charged thiolate (Fig. 1). Aspartic acid retains the charge but is unable to catalyse the dephosphorylation reaction. When tested in cells, the PRL3 C104D mutant was able to repress CNNM magnesium efflux as effectively as wild-type PRL3. In a tail-vein assay of tumour metastasis, B16 cells transformed with the C104D mutant formed as many lung nodules as the wild type.^10^ A complementary mutation, R138E, that blocks CNNM binding but not catalytic activity did not promote metastasis. These results strongly argue for the characterisation of PRLs primarily as pseudo-phosphatases.

This importance of CNNM binding has been largely ignored even though there is the widespread agreement that PRLs are poor enzymes. The extremely slow kinetics of PRLs is a direct consequence of the long lifetime of the phosphocysteine intermediate. In the absence of a mechanism to speed up hydrolysis of phosphocysteine, PRLs act in an almost stoichiometric fashion with one phosphatase molecule dephosphorylating one substrate. Studies in cells suggest that the lifetime of the phosphocysteine intermediate is several hours, which is consistent with the absence of a mechanism to promote dephosphorylation and increase activity.^5,10^

Paradoxically, although the substrates are unknown, there is good evidence for a small amount of PRL activity in cells and tissues. Western blotting of endogenous PRLs shows that the majority of PRLs are phosphorylated on cysteine, which can only occur as a result of catalysis.^5^ Indirect evidence of the importance of this phosphatase activity comes from the conservation of PRL catalytic site. Typically, pseudo-enzymes lose their catalytic activity through mutations in the active site, yet PRL phosphatase activity is conserved from tardigrades to humans.^10^ When phosphorylated, PRLs are unable to bind CNNMs, which suggests that phosphorylation of the catalytic cysteine plays a role in controlling CNNM activity. The phosphorylation of PRLs changes in response to changes in magnesium in the cellular growth medium.^5^ Magnesium deprivation leads to a slow decrease in phosphorylation while re-addition leads to rapid rephosphorylation.^10^ This naturally leads to a model for Mg^2+^ homoeostasis in which PRL phosphorylation detaches PRLs from CNNMs to stimulate Mg^2+^ efflux. Thus PRLs behave both as pseudo-phosphatases and phosphatases. They regulate CNNM proteins via direct binding but are themselves regulated through their catalytic activity.

Cysteine oxidation probably provides an additional level of regulation of the PRL–CNNM interaction. Little is known about the oxidation state in cells, but the purified PRL proteins are partially oxidised even in mildly reducing conditions.^6^ The disulphide forms of PRLs show approximately  1000-fold weaker binding to CNNM proteins and, of course, are catalytically inactive.^5^ An increase in reactive oxygen species would be expected to decrease CNNM binding and intracellular magnesium levels, possibly as a homoeostatic feedback loop to regulate mitochondrial metabolism.

Going forward, many questions remain to be answered. PRL expression levels are greatly increased in metastatic cancer and regenerating liver cells, but nothing is known about the signals that control PRL phosphorylation and oxidation. In cultured cells, PRL phosphorylation changes dramatically in response to magnesium availability but the mechanism and substrates involved are unknown. Finally, much more needs to be done to understand how changes in magnesium efflux lead to downstream effects and cancer metastasis. One point that is clear is that the PRL mutations that selectively impair phosphatase or pseudo-phosphatase activities will be valuable tools for future studies.

## Data Availability

Not applicable.
